# A synergistic future for AI and ecology

**DOI:** 10.1073/pnas.2220283120

**Published:** 2023-09-11

**Authors:** Barbara A. Han, Kush R. Varshney, Shannon LaDeau, Ajit Subramaniam, Kathleen C. Weathers, Jacob Zwart

**Affiliations:** ^a^Cary Institute of Ecosystem Studies, Millbrook, NY 12545; ^b^IBM Research - Thomas J. Watson Research Center, Yorktown Heights, NY 10598; ^c^Lamont-Doherty Earth Observatory, Columbia University, Palisades, NY 10964; ^d^U.S. Geological Survey, Water Resources Mission Area, Integrated Information Dissemination Division, San Francisco, CA 94116

**Keywords:** complex systems, ecosystems, interpretable AI, deep learning, prediction

## Abstract

Research in both ecology and AI strives for predictive understanding of complex systems, where nonlinearities arise from multidimensional interactions and feedbacks across multiple scales. After a century of independent, asynchronous advances in computational and ecological research, we foresee a critical need for intentional synergy to meet current societal challenges against the backdrop of global change. These challenges include understanding the unpredictability of systems-level phenomena and resilience dynamics on a rapidly changing planet. Here, we spotlight both the promise and the urgency of a convergence research paradigm between ecology and AI. Ecological systems are a challenge to fully and holistically model, even using the most prominent AI technique today: deep neural networks. Moreover, ecological systems have emergent and resilient behaviors that may inspire new, robust AI architectures and methodologies. We share examples of how challenges in ecological systems modeling would benefit from advances in AI techniques that are themselves inspired by the systems they seek to model. Both fields have inspired each other, albeit indirectly, in an evolution toward this convergence. We emphasize the need for more purposeful synergy to accelerate the understanding of ecological resilience whilst building the resilience currently lacking in modern AI systems, which have been shown to fail at times because of poor generalization in different contexts. Persistent epistemic barriers would benefit from attention in both disciplines. The implications of a successful convergence go beyond advancing ecological disciplines or achieving an artificial general intelligence—they are critical for both persisting and thriving in an uncertain future.

Ecological understanding is paramount for confronting multiple linked phenomena including the increased frequency of disease outbreaks, exponential losses of global biodiversity, and profound impacts of climate change. These crises share a commonality: they emerge from perturbations of complex systems whose high dimensionality underpins nonlinear dynamics that are difficult to predict. Advances in AI have the potential to transform our understanding of ecological systems (1). At the same time, ecological systems are themselves an impetus for the advancement of AI. Challenges that are commonplace in multiscale, context-dependent, and imperfectly observed ecological systems offer a panoply of problems through which AI moves closer to realizing its full potential. Predicting and purposefully managing the outcomes of perturbations to natural complex systems is a great challenge of our time, one that demands bold synergistic convergence of AI and ecological science to achieve a more holistic understanding that informs action—creating system wisdom toward a resilient future (Fig. 1).

**Fig. 1.**
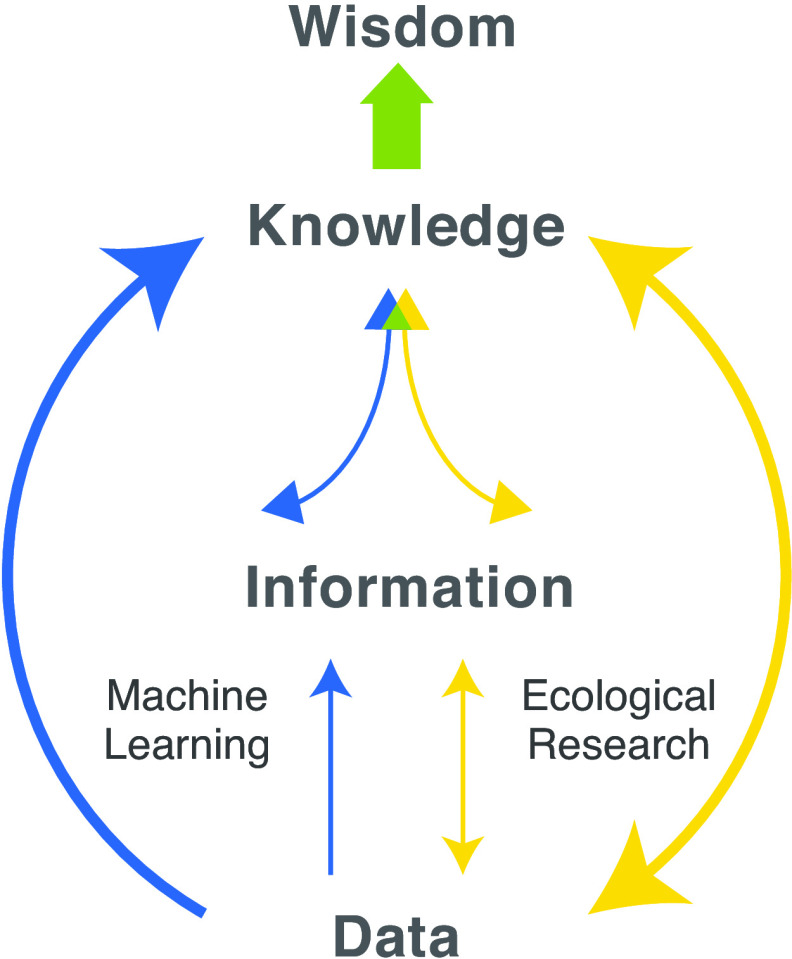
A diagram illustrating interconnections between data, information, knowledge, and wisdom. **Data** reflect raw observations or measurements (e.g., satellite data such as sea surface temperature (SST) at a specific location), while synthesizing these measurements into a meaningful form constitutes **information** (e.g., providing spatial or temporal context to SST measurements in the form of time series maps). **Knowledge** adds context, providing similar examples or comparisons to other knowledge systems (e.g., some marine organisms will experience heat stress). Finally, **wisdom** considers all of these elements as well as societal or cultural values to assess potential actions (e.g., carbon emission limits to mitigate detrimental climate warming effects). Arrows represent how machine learning (ML, blue) and ecological systems research (yellow) achieve connections within this framework. ML can transform data into information but can also bypass the information step to direct inference conveyed as knowledge. In contrast, the bidirectional arrows for ecology represent iterative feedback to the process of data collection in order to achieve knowledge through statistical modeling and hypothesis refinement. Intentional and synergistic advances in ML-AI and ecological systems science may spur greater understanding, prediction, and protection of complex systems function if we can align strengths from each discipline to explicitly identify biases and manage uncertainty and different ways of knowing, especially at information and knowledge levels.

Just as humans learn patterns from data to build intelligent thinking about a system, ML underpins modern AI, the proximate goal of which is to execute tasks or make decisions in a particular domain. Applying AI tools to ecological domains has vastly expanded our abilities to quantify phenomena that were previously unquantifiable or difficult to observe (2), and offers the possibility of faster and more accurate predictions about ecological systems. Recent examples include studying the interactions between organisms and their environments through camera and acoustic data (3–5); distilling earth systems satellite data into meaningful ecological functions (e.g., productivity) (6); analyzing animal behavior through deep learning and pose estimation (7); bioinformatics for prediction and validation of whether new viruses are capable of human infection (8); and which animal species are most likely to harbor them (9).

These are examples of "AI for ecology"—the application of existing AI tools to ecological problems. The other direction, however, of ecological science inspiring new paradigms in AI is just as important. An ultimate goal in AI research is to achieve artificial *general* intelligence (AGI) that can extrapolate and reason about other domains and systems similar to human intelligence. It is likely that AGI will need a combination of data-driven ML and new ways of representing and reasoning from diverse knowledge types to meet the challenge of trustworthy predictions about no-analog futures, such as those we anticipate on a rapidly changing planet. Meeting these challenges may be helped by a fundamental shift in how AI and ecological research propel each other.

Here, we identify a convergence on the horizon between ecological research, which has traditionally lagged behind advances in AI and computational science and AI research (Fig. 2). This convergence seeks new thought paradigms to support intelligent extrapolation to unobserved (or unobservable) systems and futures. Our argument extends well beyond the "AI for X" paradigm of simply applying AI to myriad domains (represented by "X"). A shift toward coproduced, convergent research has the potential to fuel the next generation of AI advances and ecological understanding.

**Fig. 2.**
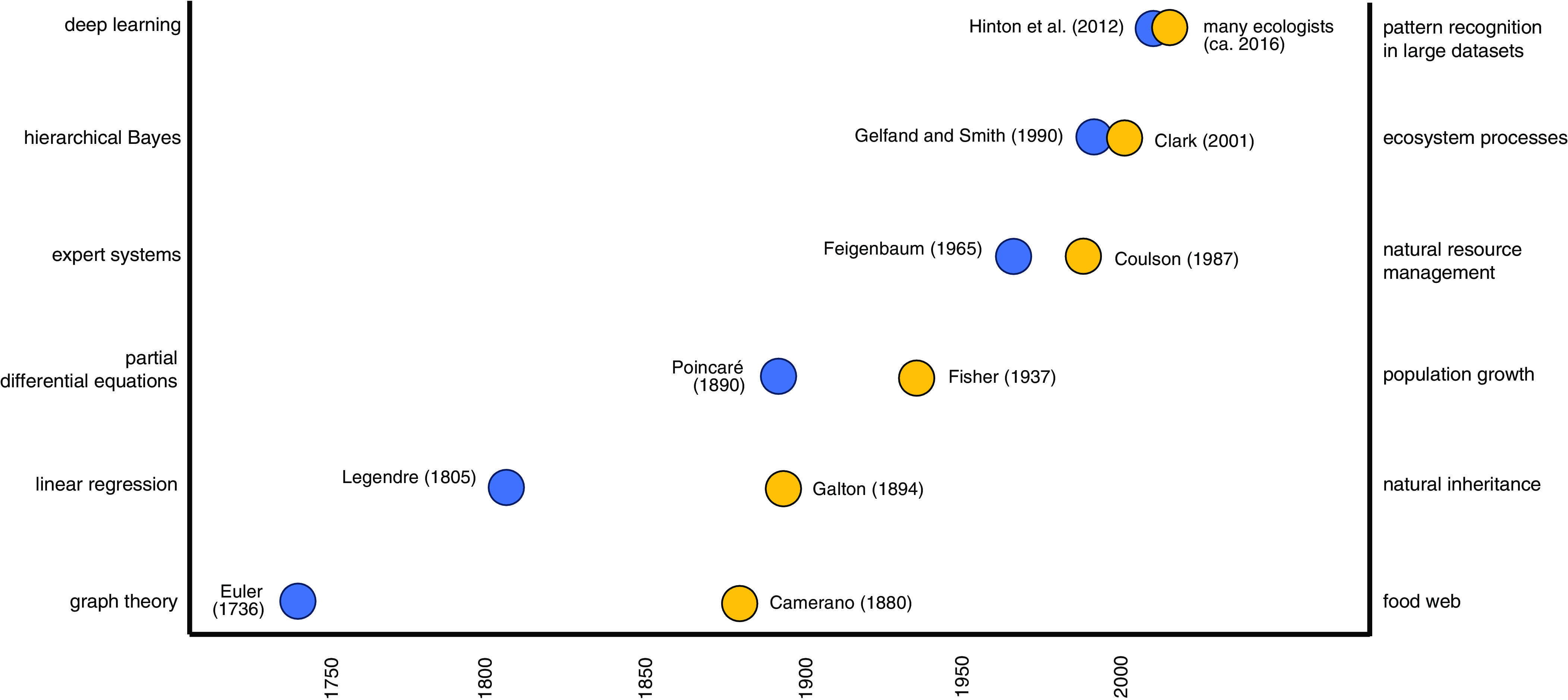
The arc of computational methods in ecological modeling (yellow) has lagged behind the development of the computational methods themselves (blue) but is close to converging. The figure is meant to be evocative, not comprehensive. It is biased toward Western scientific traditions and data points may be contested. Figure citations (10–21), with Lederberg (20) as a reference for Feigenbaum 1965, and Krizhevsky et al. (10) to accompany Hinton et al. (19).

This convergence would be helped by the amalgamation of two cultures of scientific understanding (22). In ecology, a main objective is to understand complex systems spanning the physical and the biological. This understanding is often captured in mathematical models, which reflect our hypotheses about the mechanisms that act in concert to produce observable outcomes. But ecology neither succumbs to the "unreasonable effectiveness of mathematics" (like purely physical phenomena) (23) nor to the "unreasonable effectiveness of data" (like certain biological phenomena) (24). Simple mathematical equations cannot fully capture the essence of an ecological system. Likewise, complicated ML models do not generalize well enough to capture nonlinearities from unexpected perturbations—ML models derive algorithmic understanding from data that are often about one particular system. Furthermore, because the goal of ecological research is not just prediction but phenomenological and mechanistic understanding of complex systems, ecologists use a variety of modeling techniques at different scales that together coherently capture emergent properties in a holistic system-level way that is conducive to further study (25). This approach contrasts with AI research and development, where conflicts among multiple models are not undesired because the models are only being used for prediction (which one performs best), not for explanation (why does one perform better than another) (26). For example, large language models in AI show emergent behaviors that are not present in smaller language models (27), but current techniques in AI do not explain them. The modus operandi of ecological systems research, which prioritizes understanding the mechanisms generating the data we observe, can push AI researchers to focus on methods permitting deeper understanding of the causes underlying such observed phenomena—what quantitative changes in a system lead to qualitative changes in system behavior (27).

A critical emergent behavior of ecological systems—an intelligence if you will—is their spectacular resilience to perturbation. This quality further points to ecology as an inspiration for AI, which has only experienced mixed success with imbuing resilience into the existing brittle intelligence of neuroinspired AI (28). Thus, it is not only the different and combined ways of modeling ecological systems that can help push AI research, but the ecological systems themselves that can inspire robust multiscale architectures for AI. [This echoes the synergistic relationship between quantum chemistry and quantum computing (29).]

By coproducing an interwoven research path, AI and ecological understanding are well positioned to mutually advance beyond where each discipline could arrive independently. A future where AI research development is synergistic with ecological research could advance the quest to understand complex ecological systems at temporal and spatial scales relevant to societal needs. Below, we provide an account of the current state of the art in AI research (Section 1). We briefly trace the history of ecological systems modeling up to the present use of deep neural networks (Section 2) and expand upon the opportunity for convergent research in AI and ecology. We then conjecture examples of this convergent research paradigm, exploring how the study of ecology can advance AI (Section 3), how AI is advancing ecology (Section 4), and opportunities for synergistic research to accelerate mutual discovery and advancement (Section 5). We conclude by identifying some distinct and common biases that should be addressed by AI and ecology disciplines and highlight some shared opportunities for more responsible development and deployment of AI (Section 6).

## 1. AI: The Current State of the Art

In the last 10 y, deep neural networks (also known as deep learning) have become synonymous with AI due to their powerful modeling abilities. Familiar successes include more accurate clinical diagnoses from radiological imaging and increasingly rapid analysis and decision-making used for self-driving car technology. In December 2022, ChatGPT, a language model based on deep learning, showed the rapidly expanding potential of deep learning models. Deep neural networks are a subfield of ML. ML derives its intelligence from the patterns in data, whether tabular data, time series, images, or text. The goal is to generalize the patterns from these data to new unseen data points. ML models have many forms; some are simpler, like decision trees and linear models, and others are more complex. For example, artificial neural networks are inspired by the wiring of neurons in brains and utilize a structure of layers that compute weighted dot-products fed through nonlinear functions. Deep neural networks have many layers and are trained on large datasets.

Despite the recent prominence of ML and particularly deep learning within AI, several other approaches to AI have developed in parallel and may overcome the limitations of deep learning to model complex systems and enable more resilient intelligence. One example is symbolic AI, which involves logical reasoning over knowledge graphs. A knowledge graph is different from statistical data in that it explicitly captures concepts and their semantic relationships. A graph might have "animals", "cows", "plants", and "grasses" as vertices with edges between them indicating that cows "are" animals, grasses "are" plants, and cows "eat" grasses. An AI system can then reason broader generalizations, such as that some animals eat some plants. Knowledge graphs are one type of representation used in symbolic AI. Other representations are ontologies, logical rule sets, probabilistic dependency graphs, differential equations, and analytic equations; each has reasoning algorithms built on top of them (30). This kind of knowledge and reasoning constitutes expert systems.

The present state of the art in AI is based on "foundation models" trained on unimaginably large datasets used as base models for many different tasks (31). A process of fine-tuning on a small domain-specific dataset specializes the foundation model for the task. Moreover, foundation models are being used within generative models, which are able to create new data (32), for example, to generate the sequences and molecular structures of new viral variants through which to investigate hypotheses about infection risk to humans and other animals.

In another vein, neurosymbolic AI is combining the best qualities of deep neural networks and knowledge-based symbolic methods to push beyond each method's unique limitations (33). Neurosymbolic AI systems are considered to be broader than more narrowly abled deep learning methods and are a step farther on the path toward AGI. They can help enable challenging knowledge discovery tasks (generating new hypotheses) and deal with heterogeneous and complex data of different modalities, scales, qualities, and quantities—all of which are common in ecological inquiry of complex systems. Like Bayesian statistical approaches, neurosymbolic AI can incorporate diverse modes of expert knowledge in its reasoning that are not always presented as data. It may also be more adaptable and robust than other AI approaches and may give explainable outputs that lend mechanistic insight, which is a guiding principle of ecological research (34).

## 2. Ecological Systems Modeling

Prediction in ecological systems epitomizes the difficulty in modeling complex systems more generally, which are defined by nonlinear dynamics that arise from feedbacks and dependencies that span multiple scales of time, space, and social dimensions. Because the science of ecological systems sits at the intersection of multiple mature subdisciplines, many physical and biological principles can guide our understanding about these systems—for example, the physics of hydrology, biogeochemistry, and landscape ecology (35), or the principles of fitness in population dynamics. Extracting information from observations about ecological systems ideally would consider the stochasticity and context dependence that are inherent in ecological systems. Ecologists have met this challenge with a plethora of modeling approaches, some focusing on system components (e.g., questions such as, What are the biophysical controls on the distribution and abundance of a limited resource?) and others focusing on modeling the bigger picture (e.g., How will ecological communities function differently in a rapidly changing landscape?). Improving systems-level prediction in ecology may also be fruitful for the development of novel AI, and these innovations may happen more quickly than they have in the past.

**Fig. 3.**
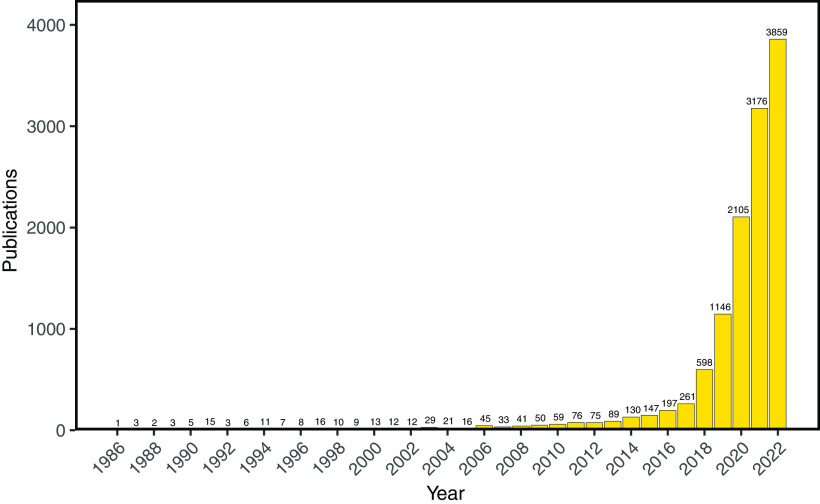
The number of papers per year for the query on Web of Science: ((TS=("artificial intelligence" OR "machine learning"))) AND WC=(Ecology OR "environmental sciences"). Certain data included herein are derived from Clarivate Web of Science (40). © Copyright Clarivate 2023. All rights reserved.

Adoption of new computational methods has generally exhibited a multiyear lag in ecological modeling (Fig. 2). Examples include graph theory (e.g., for modeling food webs), linear regression (e.g., for modeling heredity), partial differential equations (e.g., for modeling population growth), expert systems (e.g., for managing environmental decisions), hierarchical Bayesian methods (e.g., for estimating tree fecundity), and deep learning (e.g., for assessing biodiversity). The historical lag between the invention of a computational method and its integration into ecological modeling appears to be closing (36) (Fig. 2), and the use of AI in ecological modeling has greatly increased (Fig. 3). ML–based prediction has started supplementing theory-driven prediction in areas such as hydrology (37), zoonotic disease ecology (38), and forest ecology (39). However, the scope of application is still largely limited to pattern-recognition and prediction, and AI remains underutilized as a tool, for example, for synthesizing big data in ecology or for identifying and prioritizing novel hypotheses about ecological function for further investigations.

Analogous to brain-inspired breakthroughs in deep learning (41), the self-organizing properties and processes of biology are likely to hold hidden inspirations for AI system design that will be revealed by a closer coupling of AI research and ecological problems. Extending such biological inspiration offers a new lens highlighting opportunities for AI innovations that borrow from empirically observed intelligent decision-making from simple organisms, like slime molds, that defy our current notions of "intelligence" (42). Similarly, evolutionary principles that are fundamental to biology and ecology have inspired AI research. Evolutionary computing is an ecology-inspired branch of AI that applies genetic algorithms to direct the evolution of systems for applied goals, whose advances stem from in silico studies, such as directed evolution in microbes (43). Symbolic regression is another form of evolutionary computing that is currently being advanced through applications in ecology (44, 45), leading to human-interpretable functional equation models of complex ecological systems that are compositions of more primitive equations. Biodiversity measurements in ecology, which often serve as a surrogate for ecosystem complexity, are inspiring AI researchers to develop new ways to measure unwanted bias in training data (46).

We are bullish about the synergistic advances that can be made between AI and ecological research—where ecological theory has potential to advance AI research frontiers; where existing AI methods are infused into the ecological modeling of complex systems; and where coproduced AI and ecological research hold promise for critical mutual advances.

## 3. Ecology for AI: Resilience Theory

In ecology, resilience is the capacity of a system to resist or recover from perturbation. Ecological systems are resilient because the ecological function, or the roles that are played by members of the system, are both redundant and context-dependent. Understanding the resilience of our ecological systems is one of the most critical questions facing modern science. Progress in our ability to measure and make predictions about system resilience will determine the degree to which we are able to prepare for and respond to the reverberating impacts of global climate change and land use affecting the biological processes and higher-order interactions that underpin ecosystem resilience (47). Resilience theory in ecology (48) may offer clues to AI researchers for building more robust and adaptable systems involving feedback loops, redundant pathways, and satisficing behaviors that identify which fundamental principles of a system would ideally be quantified and captured in order to reproduce resilience (49). These AI systems could themselves be used to model and investigate ecological resilience. Out-of-distribution generalization and resilience to distribution shift is an active area of AI research (50). Future AI technologies that could model such context-dependent behavior may benefit from complex and nonlinear interactions with robustness built-in. Purposeful joint advances between AI research and ecology have the potential to extend general systems theory, where novel AI that is inspired and constrained by the complexities of an ecological system serve as a throughway to other domains, such as psychology or economics where prediction is similarly difficult because of complexity spanning multiple interacting scales.

## 4. AI for Ecology: Knowledge-Guided ML

Extant deep learning algorithms are data hungry and can produce predictions that are inconsistent with reality due to their architectures not including prior knowledge of the phenomena they are modeling. The growing field of knowledge-guided ML (KGML) is one way to advance both AI and ecology, especially under data-sparse conditions, which are still common in many areas of ecology (51–54). KGML seeks to inject scientific knowledge into the underpinning architecture of ML algorithms to assist resulting models in making more physically consistent predictions (55). This idea is consistent with the incorporation of prior knowledge in Bayesian statistical approaches, which have been leveraged for similar purposes in ecological studies but are often limited by both demands for data and computational costs (e.g., refs. 56 and 57). Examples of injecting knowledge guidance into ML models include customizing loss functions to obey physical laws (53, 58), using existing ML architectures [e.g., long short-term memory (LSTM) (59) or developing new ones to better represent reality [e.g., mass conserving-LSTM (60), recurrent graph networks (61), using process-based model outputs as inputs to ML models or as pretraining datasets (62), using partial differential equations representing systems in ML models (63), or embedding neural networks into hierarchical models (64). Going forward, ecological modeling may inspire more advanced architectures that include prior ontological knowledge in combination with hierarchies, physical and biological laws, and differential equations, along with decentralized and emergent training paradigms.

## 5. Accelerated Discovery through Synergies between AI and Ecology

AI systems are beginning to expand beyond pattern recognition to hypothesis generation and discovery, in part by revealing missing links among variables in high-dimensional networks that represent complex systems. These missing links represent unanticipated interactions or dependencies in system components spanning multiple scales. The diversity and breadth of the states and processes in ecological systems offer exciting potential for advancing AI capacity to identify such missing links and generate novel hypotheses.

One opportunity for synergy between AI and ecology is in a problem known as *mode collapse* in AI research, where algorithms are unable to capture the full diversity of a multimodal distribution because modeling is necessarily focused on a small number of observed modes. Ecology and AI have alternately approached this long-standing problem—diffusion models were a starting point in ecology (65) that are now showing some success in addressing this problem in generative AI (66); but ecological modeling has advanced greatly since then, with telegraph models, reaction-diffusion models, and population cycling models making continued advances to dealing with mode collapse in ecological systems. Here, coproduced research holds mutual benefits for both AI and ecology—a relevant example is the potential for AI-generated hypotheses about multimodal distributions to elucidate what is driving the bimodal peaks in the spillover transmission of Ebola virus from wildlife hosts (67). Such generative AI would benefit from the inclusion of advanced ecological modeling techniques.

Unlike many AI systems, ecological systems are understood through theory-based rules. For example, our understanding of biogeochemistry is founded on rules in physics and chemistry, geology, hydrology, and biology. Our understanding of how populations change over time is founded on evolutionary rules underpinning the concept of fitness, which is fundamental for predator–prey interactions, competition among organisms, and the structure of food webs. However, although the theory-based approach provides understanding of mechanisms (68), it may not be adequate to meet the ecological crises we face. In AI research, foundation modeling adopts a similar philosophy—that there are rules underpinning patterns and prediction—but in contrast to ecology, foundation models learn rules algorithmically by leveraging vast amounts of available data rather than through decades of applying the scientific method. Reinforcement learning is another active frontier of AI research where such rules are explicitly incorporated to generate hypotheses about how systems will evolve and stabilize over time. Thus, despite the complexity and seemingly chaotic unpredictability of ecological systems, governing rules, whether they are theory-derived or data-derived, offer anchor points from which we may observe, explain, and predict emergent properties of complex ecological systems. This approach holds particular promise for incorporating the social dimensions of complex systems, whose effects on ecological systems have historically been underrecognized and remains a transdisciplinary frontier.

## 6. Responsible AI and Ecology

The increasing dominance of industry in AI research is leading to continued improvements of products and platforms that may provide useful methods, whereas research on ethical AI and other societal considerations is comparatively neglected (69, 70). Nevertheless, calls for safe, ethical and responsible AI as well as research into methods for mitigating biases are increasing (71–73). Indigenous, feminist, decolonial, and other critical perspectives provide a grounding for such AI research (74–76), but those perspectives remain on the fringe of AI and face epistemic barriers that would need to be overcome to be incorporated into mainstream AI research (77).

Similarly, the history of ecology is rooted in colonialism; ecological protectionism was often used to justify control of the environment; and exclusionary practices continue to generate inference that is beneficial for only a privileged subset of society (78). Ecologists are more recently working to develop practices for linking diverse knowledge to better understand socioecological systems. For example, indigenous knowledge (a.k.a. traditional ecological knowledge), obtained with informed consent and with an understanding of clear reciprocal benefits to the community, has strengthened research directions in ecology, extending to conservation, responsible management, stewardship, and the relational ethics between people and nature (79). As an example, the nuanced understanding of Arctic snow and sea–ice conditions imparted by indigenous knowledge was used to guide unmanned aerial vehicles and satellite data collection. This information was used to better understand and manage ringed seals, a culturally important species in a changing climate (80).

Nuances of social and cultural structures and their integration are essential for gaining knowledge and wisdom about complex systems, as well as for responsibly affecting their future. For example, systemic racism has profoundly affected the social, ecological, and evolutionary characteristics of urban environments and is important to consider for ensuring social justice and strengthening the resilience of these systems to climate change (81). It is critical that both AI and ecology continue to expand epistemic boundaries, recognizing different ways of knowing as scientifically valid and respecting indigenous data sovereignty. In order to do this successfully, both could learn from qualitative research methodologies from social science (82) and specifically to capture “the context inextricably tied to the Indigenous epistemology, while maintaining standardized temporal and representational parameters that will make it consistent with other datasets for integration and analysis” (the hierarchical picture of Fig. 1 is contested under various epistemologies) (83). Additionally, following the CARE principles [Collective Benefit, Authority to Control, Responsibility, and Ethics, (84)] for indigenous data can help ensure that these data are useful for indigenous peoples and remain under their control, while also advancing knowledge within AI and ecology.

## 7. Looking toward Convergence

The research enterprise has been moving along disciplinary trajectories, fast approaching an important convergence between AI and the science of ecological systems. Convergent research in biomedicine underscores the unrealized potential of AI for achieving what were previously characterized as "moonshots"—cures for infectious diseases that have yet to emerge (85) and for noninfectious diseases stemming from multiple sets of interacting factors (86).

Accelerating such convergent breakthroughs would be helped by investment on multiple fronts: confronting and ameliorating biases and limitations in the data—and ways of knowing—that currently exist, considering transdisciplinary thinking and practices to bridge philosophical and ethical differences in what constitutes knowledge (87), and building trust while exploring new disciplinary languages and perspectives. Investing in such intentional convergence has the potential to yield transformative perspectives and solutions that are as unimaginable and disruptive as recent breakthroughs in chatbots and generative deep learning. In an age where rapid environmental changes pose existential risks, strategic synergies between ecological system science and AI can help propel us to better understand and potentially restore the resilience of the ecological systems upon which we depend.

## Data Availability

Literature search outputs for Fig. 3 data have been deposited in Figshare (https://doi.org/10.25390/caryinstitute.22312177) (40).
